# Clinical and Immunological Response in Dogs Naturally Infected by *L. infantum* Treated with a Nutritional Supplement

**DOI:** 10.3390/ani9080501

**Published:** 2019-07-30

**Authors:** Pietro Lombardi, Anna Teresa Palatucci, Angela Giovazzino, Vincenzo Mastellone, Giuseppina Ruggiero, Valentina Rubino, Nadia Musco, Rosalia Crupi, Monica Isabella Cutrignelli, Domenico Britti, Giuseppe Vassalotti, Giuseppe Terrazzano, Laura Cortese

**Affiliations:** 1 Department of Veterinary Medicine and Animal Productions, University of Naples Federico II, 80138 Napoli NA, Italy; 2 Department of Science, University of Basilicata, 85100 Potenza PZ, Italy; 3 Department of Translational Medical Sciences, University of Naples Federico II, 80138 Napoli NA, Italy; 4 Department of Chemical, Biological, Pharmaceutical and Environmental Sciences, University of Messina, 98122 Messina ME, Italy; 5 Department of Health Sciences, University Magna Graecia, 88100 Catanzaro CZ, Italy

**Keywords:** Leishmaniasis, dogs, nutraceuticals, immunomodulation, T helper cells, T regulatory cells

## Abstract

**Simple Summary:**

We evaluated the effects of a commercial nutraceutical supplementation in dogs naturally affected by *Leishmania infantum*. This nutraceutical supplement is proposed to be added to dog diet to foster physiological immune-response during chronic leishmaniasis. Parasite clearance is infrequently obtained by immune response in both the human and canine leishmaniasis. Pharmacological therapies frequently fail in the elimination of *L. infantum* that could have settled in deep organs, escaping immune responses and therapy. The conventional drug therapy improves clinical signs, reduces parasitemia as well as relapse of infection. This nutraceutical supplementation can improve the impaired immune response of the infected dogs to fight the disease.

**Abstract:**

The use of nutraceuticals as immunomodulators in the treatment of visceral leishmaniasis has generated interest in the current approaches to treat the disease. In this clinical and immunological study, we investigated whether the administration of a nutritional supplement mediates the immune-modulatory response in canine leishmaniosis (CL) and improves the clinical outcome of the disease. With this purpose, we analysed T lymphocyte subsets in peripheral blood (PB) of 12 dogs naturally infected by *Leishmania infantum*, following treatment with a nutritional supplement. The regulatory T (Treg) cells and the T helper (Th) 1 population were specifically evaluated. The animals underwent complete clinical examination and blood sample collection for haematological, biochemical, serological and immunological analysis before treatment (T0), one month (T30) and 3 months (T90) after the onset of the nutraceutical supplementation. We observed that nutraceutical supplementation was associated with immunomodulation of Th1 response and significant clinical improvement of the animals. No side effects were observed. Therefore, a potential supportive role for the nutraceutical supplement during canine leishmaniasis is proposed.

## 1. Introduction

Canine Leishmaniasis (CL) is a relevant endemic zoonotic disease in Mediterranean region where dogs are considered to be the main reservoirs hosts. In Europe, CL is caused by the protozoan *L. infantum* and usually transmitted by phlebotomine sand flies, although non-vectorial transmission is also reported [1,2,3]. Clinical symptomology and progression of CL is variable [4,5]. CL can range from a self-limiting disease with sub-clinical appearance [6,7], to a non-self-limiting infection with severe clinical features [4,8,9]. Subclinical or moderate infection is generally characterized by effective anti-*Leishmania* Th1 response with interferon (IFN)-γ and tumour necrosis factor (TNF)-α production that increases the leishmanicidal activity of the macrophages [10,11,12]. On the contrary, CL severe disease is associated with poor cell-mediated immune response and the occurrence of a mixed Th1 and Th2 cytokine profile [13]. Involvement of CD4^+^CD25^high^Foxp3^+^ T regulatory cells (Treg) could be of critical relevance during CL [14,15,16]. Treg regulate the recruitment and activation of immune effectors [17]. During CL, Treg activity may suppress anti-parasite immune response and exacerbate the *L. infantum* growth, favouring severe disease occurrence. On the contrary, Treg could avoid both the immune-mediated tissue damage and autoimmune processes in CL [18,19,20,21,22,23,24,25]. In this regard, we previously described the occurrence of CD8^+^CD3^+^ T and Th1 cell increase accompanied by Treg decrease in CL [14].

Meglumine antimoniate, miltefosine and aminosidine are the only registered drugs for CL in Europe [22]. Administration of Allopurinol during and after antimonial treatment avoids relapses [22,26]. Therapy with Allopurinol alone should be continued for 6 months [27] or one year [22,28,29]. The combination of miltefosine with Allopurinol and Amphotericin B represents the second and the third lines of CL therapy [22]. Efficacy of CL therapies varies from poor to good [22,30,31,32]. Majority of CL dogs clinically ameliorated after one month of CL therapy [33], although a longer period is required to achieve complete clinical recovery. Reports of incremental resistance to antimonial drugs in CL [34] has induced the discovery of new drugs or natural origin compounds for the treatment of leishmaniasis in human and dogs [35]. Moreover, preventive vaccination and topical ectoparasiticidals against sand-flies have been suggested [22].

Unbalanced diet and hyponutrition are associated with increased susceptibility to infection [36,37,38,39]. In addition, malnutrition was referred as a primary cause of immune suppression and could represent a major risk factor for the susceptibility to leishmaniosis in humans and animals [40,41,42,43]. Since immune cell homeostasis and metabolism are stringently correlated, this interdependence could open to innovative immune-modulating therapeutic approaches for the treatment of infectious diseases [44,45]. In this context, the use of plant-derived nutraceuticals may regulate immune responses [46] and improve the clinical outcome of infectious diseases in both human and dog models [47,48,49,50]. Cortese et al. [15] reported that the combination of nutraceutical pet food with conventional therapy may modulate immune responses in CL. Nutraceutical diet administration correlated with the Th1 response decrease and Treg increase, and with the amelioration of CL clinical symptoms [15]. In addition, Segarra et al. [51] reported the efficacy (marked improvement in clinical and clinicopathological signs) of a treatment with dietary nucleotides and active hexose correlated compound in addition to N-methylglucamine antimoniate in dogs with leishmaniasis. Sesquiterpene (-)-α-bisabolol has been described to be effective in regulating Th1 response and in inducing clinical improvement in CL [52].

In the present study, we evaluated the efficacy of the administration of a commercial nutraceutical supplementation in dogs naturally affected by CL. This supplement is a combination of: Krill oil and flour, *Cordyceps sinensis* L., *Gentiana Lutea* L. and *Eleutherococcus senticosus* L., and is proposed to be added to the normal diet of dogs to support the natural physiological immune-response against chronic leishmaniasis. *Cordyceps sinensis* L. has been used as a tonic for longevity, endurance and vitality [53]. Such nutraceutical modulates immune responses [54,55], inhibits tumour cell proliferation [56], enhances hepatic function [57], decreases plasma cholesterol levels [58] and exhibits hypotensive and vasorelaxant properties [59]. Krill oil, extracted from the Antarctic krill (*Euphausia superba*), has been described for its anti-oxidative effect, due to astaxanthin, which keeps intact the ω-3 polyunsaturated fatty acids (PUFA) and thereby protects them from oxidation [60]. Several studies suggest the health benefits of PUFA, in particular eicosapentaenoic acid (EPA) and docosahexaenoic acid (DHA), in terms of cardiovascular and anti-inflammatory effects [61,62,63]. *Gentiana lutea* is an ancient medicinal plant with its root (*Gentianae radix*) possessing the pro-drug. Phytochemically, it is characterized by the presence of active constituents, like secoiridoids, iridoids and xanthones. Recently, it has been found that this plant is a natural source of xanthone compounds that possess antioxidative, hypoglycemic, anti-viral, anti-bacterial and hepatoprotective activities [64,65]. *Eleutherococcus senticosus* L. (Siberian ginseng) is an “adaptogen”—a stabilizer of several physiological process to increase homeostasis and stress resistance [66]. The synergy of various herbs allows to achieve a therapeutic activity [67].

In order to identify the potential immunomodulatory properties of the above nutraceutical supplement, we analysed the immune profile of 12 *L. infantum* naturally infected dogs (Nutraceutical Supplement group, NS-group), as compared with 20 sex/age-paired healthy dogs (Control group, CTR-group) at diagnosis and during a three-month follow-up.

## 2. Materials and Methods

### 2.1. Ethical Statement

Dog owners provided formal consent to house the animals in adequate facilities, to treat their dogs with the nutritional supplement and to take samples. Ethical Animal Care and Use Committee approval was obtained (OPBA, CSV, University of Naples Federico II, prot. n. 2017/0069148). This research avoided discomfort to the animals by following animal welfare guidelines.

### 2.2. Composition of the Nutraceutical

The commercial supplement is a combination of: Krill oil 3%, dry mushrooms (*Cordyceps sinensis* L.) 2%, Krill flour 1%, Gentian (*Gentiana Lutea* L.) dry root and products obtained from the extraction of herbs (*Eleutherococcus senticosus* L.).

### 2.3. Dog Enrolment and Study Design

Twelve domesticated CL-positive dogs were enrolled in Campania region (South Italy) under the supervision of the veterinarians from the Department of Veterinary Medicine and Animal Productions of the University of Study Federico II (Naples, Italy). Criterion followed for the inclusion of the subjects in the study was (i) adult dogs in the homogenous age range of 5–7 years (to exclude age groups that might have too immature or too compromised immune systems) with laboratory confirmed positivity for leishmaniasis; (ii) clinical and pathological signs of CL; (iii) and indirect immunofluorescence antibody test (IFAT) titre ≥ 160; iv) positivity of *L. infantum* DNA in sternal bone marrow (BM) aspirate, assessed by nested polymerase chain reaction (n-PCR). The exclusion criteria followed was (i) pregnant or lactating dogs (as reported by owners and in some cases, confirmation done through abdominal ultrasonography); (ii) dogs suffering with hepatic or renal disease (haematological and biochemical analysis); (iii) dogs suffering with concomitant infective diseases (see Section 2.7); and (iv) dogs treated against leishmaniasis in the last 2 years (as reported by owners). In addition, 20 healthy dogs (12 males, 8 females, equivalent in age to sick dogs) were included as control subjects (CTR-group). Healthy dogs were enrolled considering (i) clinical health condition (neither clinical signs of leishmaniasis or other infectious diseases); (ii) no clinical–pathological abnormalities by routine laboratory tests; (iii) IFAT negative (titre ≤ 1:40); (iv) negative n-PCR; and (v) exclusion of concomitant infectious diseases as described below (see Section 2.7).

In the Nutraceutical Supplement group (NS-group), CL-positive dogs received no drug treatment for 4 weeks before commencement of the trial and were administered the nutraceutical at the dose rate of 0.5 g/kg body weight for 90 days, orally. Dogs were evaluated before treatment (T0), one month (T30) and 3 months (T90) after the nutraceutical treatment. Accurate clinical examination and the blood sampling for haematological, biochemical, serological and immunological analysis were performed at T0, T30 and T90. 

The CTR-group was observed at T0 and provided both the normal control for the clinical parameters and the normality range for immune cells in our trial to be compared with NS-group.

*Leishmania* DNA n-PCR detection was performed on sternal BM aspirates in enrolled dogs at T0.

Nutraceutical supplementation was regularly added to pet food by the same dog owners before the diet administration. Diet satisfied the nutritional requirement of adult dogs in both CTR and NS groups. All dogs were subjected to ecto- and endoparacitidal treatment before the commencement of the study.

### 2.4. Clinical Evaluation of Dogs

Clinical assessment of leishmaniasis was done by veterinarians according to the criteria developed by da Silva et al. [68]:

Systemic signs—Attitude: active (0), apathetic (1); fever: absence (0), presence (1); lameness: absence (0), presence (1); nutritional status: normal (0), thin (1), cachectic (2); lymph nodes: normal (0), enlarged (1); mucosal colour: normal (0), pale (1); bleeding: absence (0), presence (1).

Cutaneous signs—Bristles: good (0), regular (1), bad/opaque (2); ear/nasal hyperkeratosis: absence (0), presence (1); nails: normal (0), long/onychogryphosis (1); skin lesion: absence (0), presence (1), ulcer (2); muzzle depigmentation: absence (0), presence (1); alopecia: absence (0), presence (1).

Ocular signs—Blepharitis: absence (0), presence (1); keratoconjunctivitis: absence (0), serous (1), mucopurulent (2).

All the individual scores were awarded to obtain a total sign-based score ranging between 0 and 19 (15 signs). Occurrence of other illnesses was monitored during the trial to rule out any positive dog. The clinical scores (CS) were obtained prior to treatments (T0) and at 1 (T30) and 3 months (T90).

### 2.5. Blood Sample Collection

Ten millilitres of peripheral blood (PB) were collected by jugular venepuncture after 12 h of fasting. Total PB amount was divided into three fractions. Complete blood count (CBC) was performed within 30 min from the collection using semi-automatic cell counter (Genius S, SEAC Radim Florence, Italy). Thrombocytopenia or evidence of platelet clumping was evaluated by May–Grünwald–Giemsa-stained blood smears. Blood chemistry analyses was performed on serum aliquots by automatic biochemical analyser (AMS Autolab, Diamond Diagnostics, Holliston, MA, USA) using reagents from Spinreact (Girona, Spain) to determine: blood urea nitrogen (BUN), creatinine, aspartate amino transferase (AST), alanine-aminotransferase (ALT), gamma-glutamyltransferase (GGT), alkaline phosphatase (ALP), total cholesterol, triglycerides, albumin and total serum proteins (TP). Serum protein electrophoresis was also evaluated.

In addition, interferon-γ was assayed in serum using Elisa kits (Genorise, Philadelphia, PA, USA).

### 2.6. Serological and Molecular Assays

Presence of anti-*Leishmania* antibodies was detected by indirect immunofluorescence antibody test (IFAT) using *L. infantum* promastigotes (WHO reference strain MHOM/TN/1980/IPT-1) as described [69]. The cut-off dilution was set at 1:160.

*E. canis* positivity was evaluated by IFAT using *E. canis* antigen in DH82 cells with a cut-off titre of 1:80.

The sternal bone marrow aspirate for *Leishmania* and *E. canis* DNA detection by n-PCR was performed as described [70,71].

### 2.7. Diagnostic Procedure

Diagnosis of CL in all dogs with clinical or clinical–pathological signs attributable to the infection was always confirmed by anti-*Leishmania* antibody titres (≥1:160) and positive molecular diagnosis. IFAT was evaluated three times (T0, T30 and T90) in NS-group.

Negative IFAT (≤1:80) and PCR, combined with the absence of clinical signs on physical examination, characterized the dogs to be included in the CTR group. 

In CTR and NS-groups, dogs detected positive for *Ehrlichia canis*, *Anaplasma phagocytophilum morulae*, *Babesia canis* trophozoites and microfilariae in peripheral blood smears were excluded from the group. Ehrlichiosis was also evaluated as described above in Section 2.6. *Dirofilaria immitis* infection was detected by Snap Canine Combo Heartworm Antigen Antibody Test (IDEXX).

### 2.8. Monoclonal Antibodies, Immunofluorescence, Flow Cytometry and Cell Culture

T cells and Treg cells were analysed by immune-fluorescence and flow cytometry (two-laser equipped FACScalibur apparatus and the CellQuest analysis software, (Becton Dickinson, Franklin Lakes, NJ, USA). Immune-fluorescence staining was performed by Fluorescin isothiocyanate (FITC), Phycoerythrin (PE), Cy-chrome and Allophycocyanin (APC) labelled monoclonal antibodies (mAbs) against dog CD3 (Clone CA17.2A12), CD4(Clone YKIX302.9), CD8 (Clone YCATE55.9), CD45 (clone CA12.10C12), IFN-γ (Clone CC302), IL-4 (Clone CC303) and isotype-matched controls (Serotec Ltd, London, UK). Intracellular Foxp3 was evaluated by a cross-reactive murine anti-FoxP3 mAb (Clone FJK-16 s, eBioscience, San Diego, CA, USA) and commercial detection Kit (FoxP3 Staining Set, eBioscience). Tregs were detected as per established protocols [14,15,72]. Peripheral blood mononuclear cells (PBMC) were purified with density gradient, cultured in RPMI 1640 (Biochrom GmbH Berlin, Germany), supplemented with 5% heat inactivated foetal bovine serum and 2 mM glutamine (Biochrom) at 37 °C in 5% CO2/95% air and incubated overnight with Phorbol myristate acetate (PMA) and Ionomycin (Sigma-Aldrich, St. Louis, MO, USA), in the presence of 5 μg/ml of Brefeldin-A (Sigma-Aldrich, St. Louis, MO, USA), to analyse IFN-γ production [14,15,73,74]. IFN-γ intracellular staining was obtained using a fixation/permeabilization kit (eBioscience). Forward Scatter (FSC) and Side Scatter (SSC) parameter strategy was applied to all analysed samples [14,15]. 

### 2.9. Statistical Analysis

Blood count, blood biochemistry and inflammatory parameters were compared by General Linear Model as:

yijk = μ + Gi + Tj + G × Tij + εijk

where: y is the dependent variable, μ is the mean, G is the group effect (i = NS, CTR), T is the time effect (j = 0, 30, 60, 90), G × T is the first level of interaction and ε is the error effect.

When significant differences were found in the ANOVA, means were compared using Tukey’s test.

Clinical signs were analysed by non-parametric Wilcoxon signed-rank test. All the statistical procedures were performed used JMP software version n. 9 (SAS Institute, Cary, NC, USA). Immunological parameters were evaluated by Wilcoxon matched pairs test (Graph-Pad Prism Inc, San Diego, CA, USA).

## 3. Results

### 3.1. Clinical and Laboratory Evaluation

All the enrolled sick dogs were symptomatic. A progressive improvement in the clinical response was observed in NS-group during the 3 months of follow-up period. No mortality was observed in the CTR and NS-group during the study. Clinical recovery was considered when a reduction in the clinical score was observed during the study. Before treatment with the nutraceutical supplement (T0), the mean clinical score in infected dogs was 6.0 (range 2–9), while the enrolled dogs showed an overall and progressive clinical improvement during follow-up (T30 mean score: 4.8, range: 1–8; T90 mean score: 3.1, range: 1–6) (Table 1). Notably, IFAT titres showed a progressive reduction (Table 1).

All haematological and biochemical parameters were within the normal range for adult dogs and no signs of adverse effects were observed after clinical examination, demonstrating that supplementation was well tolerated. Haematocrit (HT), mean corpuscular volume (MCV) and mean corpuscular haemoglobin (MCH) decreased significantly (*p* < 0.01) while mean corpuscular haemoglobin concentration (MCHC) increased significantly (*p* < 0.01) in NS-group after 90 days of treatment. White blood cells (WBC) also decreased significantly (*p* < 0.05) at day 90. Aspartate amino transferase (AST) and Blood Urea Nitrogen (BUN) resulted significantly (*p* < 0.01) higher at day 90 whereas Gamma Glutamyl Transferase (GGT) significantly increased at day 30 but decreased at day 90 compared to day 0. Beta2 and gamma globulins significantly decreased at both 30 and 90 days (*p* < 0.05 and *p* < 0.01, respectively). Interferon-γ decreased at both 30 and 90 days (*p* < 0.05) in NS-group compared to CTR-group.

### 3.2. Immune Phenotype Analysis

**Figure 1:**
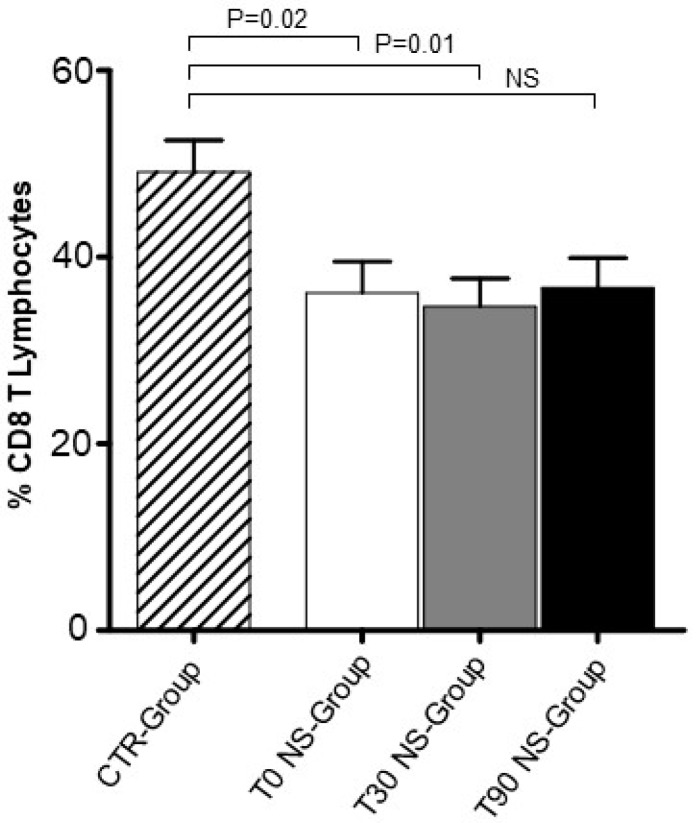
CD8 T lymphocyte percentage in NS-Group. Significant decrease of CD8 T lymphocyte percentage between NS-Group and CTR-Group was observed at T0 (*p* = 0.02) and T30 (*p* = 0.01). CD8 T lymphocyte percentage appeared to be recovered at T90 when compared to T0 and T30 and similar to the level expressed in healthy dogs. Referred values indicate results obtained in CTR-Group (dashed column), in NS-Group dogs at T0 (white column), T30 (grey column), and T90 (black column), as indicated. Wilcoxon matched-pairs signed-rank test was used for the statistical analysis NS means not significant difference.

**Figure 2:**
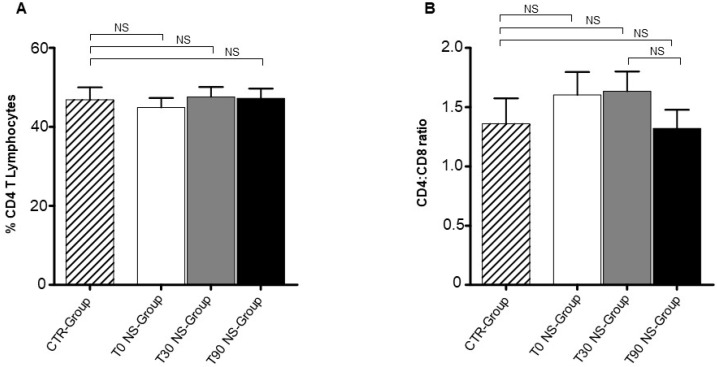
CD4 T lymphocyte percentage and CD4:CD8 cell ratio in NS-Group. CD8 T lymphocyte percentage, CD4–CD8 T cell ratio (**A**) and CD4 T lymphocyte percentage (**B**) appeared unmodified in NS-Group at T30 and T90. Referred values indicate results obtained in CTR-Group (dashed column), in NS-Group dogs at T0 (white column), T30 (grey column) and T90 (black column). Wilcoxon matched-pairs signed-rank test was used for the statistical analysis. NS means not significant difference.

**Figure 3:**
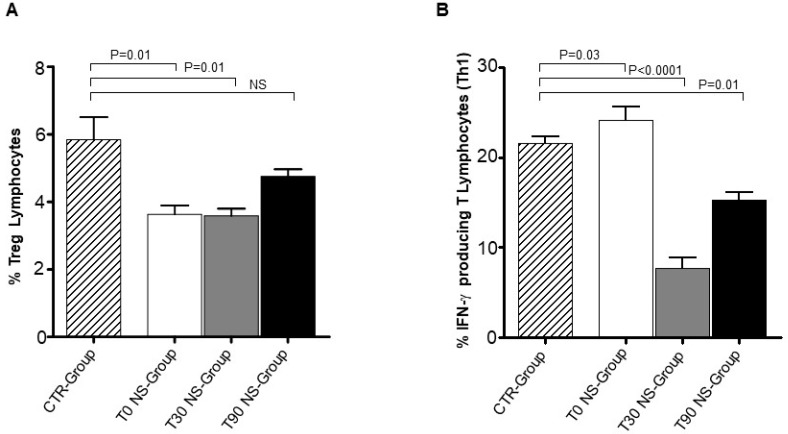
Treg lymphocyte percentage and IFN-γ producing CD3 T Lymphocytes in NS-Group. (**A**) shows the percentage reduction of Treg cells of sick dogs was observed at T0 (*p* = 0.01) and T30 (*p* = 0.01). Recovery of Treg was observed only at T90 in NS-Group. (**B**) NS-Group showed an increase of IFN-γ producing CD3 T Lymphocyte percentage at T0 as compared with CTR-Group. Nutraceutical supplementation appeared to decrease IFN-γ production at T30 and T90 in NS-Group. Referred values indicate results obtained in CTR-Group (dashed column), in NS-Group dogs at T0 (white column), T30 (grey column), and T90 (black column). Wilcoxon matched-pairs signed-rank test was used for the statistical analysis. NS means not significant difference.

We analysed by Flow Cytometry the CD8 T lymphocyte percentage in NS-Group. We observed a moderate but significant decrease of CD8 T lymphocyte percentage between NS-Group and CTR-Group at T0 (*p* = 0.02) and T30 (*p* = 0.01) (Figure 1). CD8 T lymphocyte percentage appeared to be slightly recovered at T90 when compared to T0 and T30 and became similar to the level expressed in CTR dogs. This occurrence suggests that nutraceutical supplementation was able to normalize CD8 T cell level in NS-Group. In contrast, CD4 T lymphocyte percentage unchanged in NS-Group upon nutraceutical supplementation (Figure 2B). Notably, the moderate decrease of CD8 T lymphocyte percentage and the unchanged CD4 T lymphocyte percentage could explain why the CD4:CD8 T cell ratio remained substantially unmodified in NS-Group at T30 and T90 (Figure 2A). It is noteworthy that, although the differences are not significant, the CD4–CD8 ratio appeared to be increased at T0 and T30 when compared to the CTR-Group, while it seemed to be normalized at T90 and similar to the CD4–CD8 ratio expressed in the CTR-Group. Treg percentage in the NS-Group and CTR-Group revealed a significant reduction at T0 (*p* = 0.01) and T30 (*p* = 0.01) (Figure 3A). Treg percentage restored only at T90 in NS-Group dogs and became similar to CTR-Group (Figure 3A). This result strongly suggests that Treg restoration could be correlated to the nutraceutical supplement administration.

We evaluated whether nutraceutical supplement administration could modify the Th1 activity in CL dogs. Th1 cell percentage increased in NS-Group at T0 compared with CTR-Group dogs (*p* = 0.03) (Figure 3B). This result is in accordance with our previous study on CL [14]. Nutraceutical supplementation administration appears to be associated with Th1 cell decrease at T30 (*p* < 0.0001) and T90 (*p* = 0.01) in NS-Group. This evidence was also confirmed by ELISA approach in serum CL dogs, since the IFN-γ detection changed from T0 (260.32 pg/mL) to T30 (196.25 pg/mL) and T90 (157.06 pg/mL) in NS-Group.

## 4. Discussion

Here, we described a significant improvement in clinical conditions in CL dogs treated with a nutraceutical supplement. Moreover, IFAT titres showed a progressive reduction during the follow up even if the use of antibody levels to assess clinical improvement is controversial [75,76,77]. 

No signs of adverse effects were observed after clinical examination, showing that supplementation was well tolerated. Notably, some significant differences in biochemical parameters were detected. In particular, AST was higher in NS-group than in CTR after 30 (35.8 + 8.0 U/L) and 90 (46.8 + 6.6 U/L) days of treatment vs. day 0 (37.1 + 8.2 U/L), but its levels were still comprised within physiological range. Moreover, in case of liver damage, increases in AST parallel those in ALT, which is considered a much more liver-specific marker [78], but ALT did not change after the supplement administration, thus excluding even a liver overload.

Likewise, BUN was higher in the NS group than in CTR at days 30 (35.5 + 4.4 U/L) and 90 (40.0 + 10.1 U/L) compared to day 0 (29, 7 + 7.6 U/L), also in this case in the physiological range. During leishmaniasis the kidney is primarily damaged [79]. Thus, the BUN dosage can provide useful information on the status of the kidneys. However, its prognostic value is closely related to creatinine levels [80], which were not influenced in the NS group. Therefore, the detected increases of AST and BUN have a limited diagnostic value in the NS-group dogs. In light of the clinical, biochemical and serological results, our study highlighted an improvement in the condition of CL dogs after 3 months of treatment with oral nutraceutical supplementation (NS group).

Ingredients present in the nutraceutical supplement were described to exert anti-oxidative and anti-inflammatory effects, cardiovascular benefits, hypoglycemic, anti-viral, anti-bacterial and hepatoprotective responses. *C. sinensis* L. activates murine macrophages responsible of the production of a variety of pro-inflammatory cytokines and IFN-γ synergizes with such nutraceutical in order to amplify this response [81]. *C. sinensis* reduces the incidence of diabetes, which is due to an increase in the portion of Treg cells/Th17 in the spleen and pancreatic lymph nodes [82]. Non-obese diabetic (NOD) mice treated with *C. sinensis* extract showed slowed disease development. However, in peripheral lymph nodes, treatment with *C. sinensis* extract increases the frequency of IFN-γ and Treg cells producing Th1 cells. Moreover, Chen et al. [83] showed significantly reduced CD4^+^ T cells and increased percentages of CD8^+^ T cells in peripheral blood mononuclear cells (PBMC) after *C. sinensis* administration in mice affected by lupus-prone autoimmune.

Based on in vitro data, the anti-inflammatory activity arising from *Gentiana lutea* L. is represented by the ability to inhibit myeloperoxidase enzymes, which are released during degranulation of neutrophils and monocytes. Myeloperoxidase upregulation is known to contribute to the development of inflammatory and immune-mediated complications [84].

Treg cells have been found to play a critical role to maintain self-tolerance and to prevent autoimmune diseases. Moreover, after nutraceutical administration we observed a slight restoration of Treg cells, whose low level has been associated with chronic CL [14,15]. Indeed, Treg levels appear to be increased upon treatment and become similar to those of control dogs after 90 days (T90) of nutraceutical administration. Notably, IFN-γ production progressively decreased in NS-group dogs.

Nutraceutical supplementation seems to specifically act on Treg and Th1 lymphocytes, since other type of T cells appear to be unaffected or only moderately decreased. In this regard, the unaltered T cell effector activity could ensure immune response against the parasite, while Treg increase and the modulation of Th1 inflammatory response could preserve tissues from the immune-mediated damages occurring in CL [15,18,19,21,22,23,24,25]. Moreover, the nutraceutical-induced immuno-modulation could also improve the overall clinical conditions of sick dogs due to the reduction of the inflammatory context in the pathophysiology associated with CL, such as immune-mediated thrombocytopenia [14]. Taken in all, our results showed that this peculiar nutraceutical supplement could modulate Th1 immune response and significantly improve clinical amelioration of the infected animals.

## 5. Conclusions

Nutraceuticals as a dietary supplement appear to be of tangible benefit in the clinical management of dogs affected with chronic CL. In fact, the clinical improvement and a beneficial modulation of the immune response are observed in this clinical trial. In particular, the reduction of potential adverse inflammatory events, mediated by the exacerbated INF-γ secretion and Treg percentage decreasing usually observed in CL [15,18,19,21,22,23,24,25], may be limited by the use of those nutraceuticals able to reduce the pro-inflammatory cytokine production and to increase Treg percentage. Nutraceutical supplementation appears that are able to improve the immune-modulatory response in the clinical recovery of CL dogs, independently of the conventional anti-CL treatment. The present study does not point to the replacement of CL with this nutraceutical supplementation, but suggests its use as an adjunct therapy to the conventional medical treatment of chronic canine leishmaniasis. In this regard, this study highlighted that nutraceutical supplement administration is associated with Th1 immune response modulation and significant clinical improvement of the infected animals.

## 6. Limitations

This study has some limitations: (1) No evaluation was performed to establish which ingredient is the most active in the complex of employed supplementation; (2) Absence of control group of dogs treated with other anti-oxidant and other extracts of plants, different from those here employed; (3) Absence of control group of dogs treated with conventional anti-leishmanial therapy; (4) No blinded study was conducted; (5) The trial has been performed only in 90 days-follow-up; (6) The limited sample size (12 dogs).

## Figures and Tables

**Table 1:** Evolution of clinical score (CS) and immunofluorescence antibody test (IFAT) titres in dogs belonging to NS group at time 0 (T0) and after 1 (T30) and 3 months (T90) of nutraceutical supplementation.

NS Group Dogs	T0	T30	T90
**1** CS	3	1	1
IFAT	1:1280	1:1280	1:320
**2** CS	9	8	6
IFAT	1:640	1:640	1:80
**3** CS	7	6	2
IFAT	1:640	1:640	1:320
**4** CS	7	6	5
IFAT	1:1280	1:640	1:160
**5** CS	6	6	4
IFAT	1:640	1:320	1:160
**6** CS	7	6	4
IFAT	1:320	1:320	1:40
**7** CS	5	5	3
IFAT	1:1280	1:640	1:1280
**8** CS	9	6	2
IFAT	1:640	1:640	1:320
**9** CS	7	3	3
IFAT	1:320	1:160	1:320
**10** CS	6	3	3
IFAT	1:320	1:160	1:160
**11** CS	5	2	2
IFAT	1:160	1:160	1:80
**12** CS	3	3	2
IFAT	1:640	1:640	1:160

## References

[1] Rosypal A.C., Troy G.C., Zajac A.M., Frank G., Lindsay D.S. (2005). Transplacental Transmission of a North American Isolate of Leishmania infantum in an Experimentally Infected Beagle. J. Parasitol..

[2] De Freitas E., Melo M.N., Da Costa-Val A.P., Michalick M.S.M. (2006). Transmission of Leishmania infantum via blood transfusion in dogs: Potential for infection and importance of clinical factors. Veter-Parasitol..

[3] Silva F.L., Oliveira R.G., Silva T.M., Xavier M.N., Nascimento E.F., Santos R.L., Byndloss M. (2009). Venereal transmission of canine visceral leishmaniasis. Veter-Parasitol..

[4] Baneth G., Koutinas A.F., Solano-Gallego L., Bourdeau P., Ferrer L. (2008). Canine leishmaniosis—New concepts and insights on an expanding zoonosis: Part one. Trends Parasitol..

[5] Maia C., Campino L. (2012). Cytokine and Phenotypic Cell Profiles of Leishmania infantum Infection in the Dog. J. Trop. Med..

[6] Alvar J., Canavate C., Molina R., Moreno J., Nieto J. (2004). Canine leishmaniasis. Adv. Parasitol..

[7] Lombardo G., Pennisi M.G., Lupo T., Chicharro C., Solano-Gallego L. (2014). Papular dermatitis due to Leishmania infantum infection in seventeen dogs: Diagnostic features, extent of the infection and treatment outcome. Parasites Vectors.

[8] Ciaramella P., Oliva G., De Luna R., Ambrosio R., Cortese L., Persechino A., Gradoni L., Scalone A. (1997). A retrospective clinical study of canine leishmaniasis in 150 dogs naturally infected by Leishmania infantum. Veter-Rec..

[9] Baneth G., Green C.E. (2006). Leishmaniosis. Infectious Diseases of the Dog and Cat.

[10] Zafra R., Jaber J., Pérez-Écija R., Barragan A., Martinez-Moreno A., Pérez J. (2008). High iNOS expression in macrophages in canine leishmaniasis is associated with low intracellular parasite burden. Veter-Immunol. Immunopathol..

[11] Carrillo E., Moreno J. (2009). Cytokine profiles in canine visceral leishmaniasis. Veter-Immunol. Immunopathol..

[12] Abbehusen M.M.C., Almeida V.D.A., Solcà M.D.S., Pereira L.D.S., Costa D.J., Gil-Santana L., Bozza P.T., Fraga D.B.M., Veras P.S.T., Dos-Santos W.L.C. (2017). Clinical and immunopathological findings during long term follow-up in Leishmania infantum experimentally infected dogs. Sci. Rep..

[13] Alexander J., Brombacher F. (2012). T Helper1/T Helper2 Cells and Resistance/Susceptibility to Leishmania Infection: Is This Paradigm Still Relevant?. Front. Immunol..

[14] Cortese L., Annunziatella M., Palatucci A.T., Rubino V., Piantedosi D., Di Loria A., Ruggiero G., Ciaramella P., Terrazzano G. (2013). Regulatory T cells, Cytotoxic T lymphocytes and a TH1 cytokine profile in dogs naturally infected by Leishmania infantum. Res. Veter-Sci..

[15] Cortese L., Annunziatella M., Palatucci A.T., Lanzilli S., Rubino V., Di Cerbo A., Centenaro S., Guidetti G., Canello S., Terrazzano G. (2015). An immune-modulating diet increases the regulatory T cells and reduces T helper 1 inflammatory response in Leishmaniosis affected dogs treated with standard therapy. BMC Veter-Res..

[16] Sakaguchi S. (2003). The origin of FOXP3-expressing CD4^+^ regulatory T cells: Thymus or periphery. J. Clin. Investig..

[17] Sakaguchi S. (2005). Naturally arising Foxp3-expressing CD25^+^CD4^+^ regulatory T cells in immunological tolerance to self and non-self. Nat. Immunol..

[18] Kharazmi A., Rezai H., Fani M., Behforouz N. (1982). Evidence for the presence of circulating immune complexes in serum and C3b and C3d on red cells of kala-azar patients. Trans. R. Soc. Trop. Med. Hyg..

[19] Ferrer L., Kirk R.W., Bonagura J.D. (1992). Leishmaniosis. XI Current Veterinary Therapy.

[20] Pelagalli A., Ciaramella P., Lombardi P., Pero M.E., Cortese L., Corona M., Oliva G., Avallone L. (2004). Evaluation of Adenosine 5’-diphosphate (ADP)- and Collagen-induced Platelet Aggregation in Canine Leishmaniasis. J. Comp. Pathol..

[21] Terrazzano G., Cortese L., Piantedosi D., Zappacosta S., Di Loria A., Santoro D., Ruggiero G., Ciaramella P. (2006). Presence of anti-platelet IgM and IgG antibodies in dogs naturally infected by Leishmania infantum. Veter-Immunol. Immunopathol..

[22] Solano-Gallego L., Koutinas A., Miró G., Cardoso L., Pennisi M., Ferrer L., Bourdeau P., Oliva G., Baneth G. (2009). Directions for the diagnosis, clinical staging, treatment and prevention of canine leishmaniosis. Veter-Parasitol..

[23] Cortese L., Piantedosi D., Ciaramella P., Pero M.E., Sica M., Ruggiero G., Terrazzano G., Mastellone V. (2009). Secondary immune-mediated thrombocytopenia in dogs naturally infected by Leishmania infantum. Veter-Rec..

[24] Cortese L., Pelagalli A., Piantedosi D., Cestaro A., Di Loria A., Lombardi P., Avallone L., Ciaramella P. (2009). Effects of therapy on haemostasis in dogs infected with Leishmania infantum, Ehrlichia canis, or both combined. Veter-Rec..

[25] Cortese L., Terrazzano G., Piantedosi D., Sica M., Prisco M., Ruggiero G., Ciaramella P. (2011). Prevalence of anti-platelet antibodies in dogs naturally co-infected by Leishmania infantum and Ehrlichia canis. Veter-J..

[26] Miró G., Cardoso L., Pennisi M.G., Oliva G., Baneth G. (2008). Canine leishmaniosis – new concepts and insights on an expanding zoonosis: Part two. Trends Parasitol..

[27] Miró G., Oliva G., Cruz I., Cañavate C., Mortarino M., Vischer C., Bianciardi P. (2009). Multicentric, controlled clinical study to evaluate effectiveness and safety of miltefosine and allopurinol for canine leishmaniosis. Veter-Dermatol..

[28] Torres M., Bardagí M., Roura X., Zanna G., Ravera I., Ferrer L. (2011). Long term follow-up of dogs diagnosed with leishmaniosis (clinical stage II) and treated with meglumine antimoniate and allopurinol. Veter-J..

[29] Paradies P., Sasanelli M., Amato M.E., Greco B., De Palo P., Lubas G. (2012). Monitoring the reverse to normal of clinico-pathological findings and the disease free interval time using four different treatment protocols for canine leishmaniosis in an endemic area. Res. Veter-Sci..

[30] Noli C., Auxilia S.T. (2005). Treatment of canine Old World visceral leishmaniasis: A systematic review. Veter-Dermatol..

[31] Ribeiro R.R., Moura E.P., Pimentel V.M., Sampaio W.M., Silva S.M., Schettini D.A., Alves C.F., Melo F.A., Tafuri W.L., Demicheli C. (2008). Reduced Tissue Parasitic Load and Infectivity to Sand Flies in Dogs Naturally Infected by Leishmania (Leishmania) chagasi following Treatment with a Liposome Formulation of Meglumine Antimoniate. Antimicrob. Agents Chemother..

[32] Miró G., Gálvez R., Fraile C., A Descalzo M., Molina R. (2011). Infectivity to Phlebotomus perniciosus of dogs naturally parasitized with Leishmania infantum after different treatments. Parasites Vectors.

[33] Pennisi M.G., De Majo M., Masucci M., Britti D., Vitale F., Del Maso R. (2005). Efficacy of the treatment of dogs with leishmaniosis with a combination of metronidazole and spiramycin. Veter-Rec..

[34] Gramiccia M., Gradoni L., Orsini S. (1992). Decreased sensitivity to meglumine antimoniate (Glucantime) of Leishmania infantum isolated from dogs after several courses of drug treatment. Ann. Trop. Med. Parasitol..

[35] Schmidt T., Khalid S., Romanha A., Alves T., Biavatti M., Brun R., Da Costa F., De Castro S., Ferreira V., De Lacerda M. (2012). The Potential of Secondary Metabolites from Plants as Drugs or Leads Against Protozoan Neglected Diseases—Part I. Curr. Med. Chem..

[36] Ambrus J.L., Ambrus J.L. (2004). Nutrition and infectious diseases in developing countries and problems of acquired immunodeficiency syndrome. Exp. Biol. Med..

[37] Katona P., Katona-Apte J. (2008). The Interaction between Nutrition and Infection. Clin. Infect. Dis..

[38] Ovchinnikov R.S., Farhadi S. (2018). The relationship between nutrition and infectious diseases: A review. Biomed. Biotechnol. Res. J. (BBRJ).

[39] Hennig B., Petriello M.C., Gamble M.V., Surh Y.J., Kresty L.A., Frank N., Rangkadilok N., Ruchirawat M., Suk W.A. (2018). The role of nutrition in influencing mechanisms involved in environmentally mediated diseases. Rev. Environ. Heal..

[40] Anstead G.M., Chandrasekar B., Zhao W., Yang J., Perez L.E., Melby P.C. (2001). Malnutrition Alters the Innate Immune Response and Increases Early Visceralization following Leishmania donovani Infection. Infect. Immun..

[41] Malafaia G. (2009). Protein-energy malnutrition as a risk factor for visceral leishmaniasis: A review. Parasite Immunol..

[42] Carrillo E., Jiménez M.Á., Sánchez C., Cunha J.M., Martins C.M., Sevá A.D.P., Moreno J. (2014). Protein Malnutrition Impairs the Immune Response and Influences the Severity of Infection in a Hamster Model of Chronic Visceral Leishmaniasis. PLoS ONE.

[43] Mengesha B., Endris M., Takele Y., Mekonnen K., Tadesse T., Feleke A., Diro E. (2014). Prevalence of malnutrition and associated risk factors among adult visceral leishmaniasis patients in Northwest Ethiopia: A cross sectional study. BMC Res. Notes.

[44] Matarese G., Moschos S., Mantzoros C.S. (2005). Leptin in immunology. J. Immunol..

[45] Gerriets V.A., Rathmell J.C. (2012). Metabolic Pathways in T Cell Fate and Function. Trends Immunol..

[46] Andlauer W., Fürst P. (2002). Nutraceuticals: A piece of history, present status and outlook. Food Res. Int..

[47] Khoo C., Cunnick J., Friesen K., Gross K.L., Wedekind K., Jewell D.E. (2005). The role of supplementary dietary antioxidants on immune response in puppies. Veter-Ther. Res. Appl. Veter-Med..

[48] Chew B.P., Mathison B.D., Hayek M.G., Massimino S., Reinhart G.A., Park J.S. (2011). Dietary astaxanthin enhances immune response in dogs. Veter-Immunol. Immunopathol..

[49] Marsella R., Santoro D., Ahrens K. (2012). Early exposure to probiotics in a canine model of atopic dermatitis has long-term clinical and immunological effects. Veter-Immunol. Immunopathol..

[50] Colitti M., Gaspardo B., Della Pria A., Scaini C., Stefanon B. (2012). Transcriptome modification of white blood cells after dietary administration of curcumin and non-steroidal anti-inflammatory drug in osteoarthritic affected dogs. Veter-Immunol. Immunopathol..

[51] Segarra S., Miró G., Montoya A., Pardo-Marin L., Boqué N., Ferrer L., Cerón J. (2017). Randomized, allopurinol-controlled trial of the effects of dietary nucleotides and active hexose correlated compound in the treatment of canine leishmaniosis. Veter-Parasitol..

[52] Corpas-López V., Merino-Espinosa G., Acedo-Sánchez C., Díaz-Sáez V., Navarro-Moll M.C., Morillas-Márquez F., Martín-Sánchez J. (2018). Effectiveness of the sesquiterpene (-)-α-bisabolol in dogs with naturally acquired canine leishmaniosis: An exploratory clinical trial. Vet. Res. Commun..

[53] Zhu J.S., Halpern G.M., Jones K. (1998). The Scientific Rediscovery of an Ancient Chinese Herbal Medicine: Cordyceps sinensis Part I. J. Altern. Complement. Med..

[54] Yang L.Y., Chen A., Kuo Y.C., Lin C.Y. (1999). Efficacy of a pure compound H1-A extracted from Cordyceps sinensis on autoimmune disease of MRL lpr/lpr mice. J. Lab. Clin. Med..

[55] Kuo Y.C., Tsai W.J., Wang J.Y., Chang S.C., Lin C.Y., Shiao M.S. (2001). Regulation of bronchoalveolar lavage fluids cell function by the immunomodulatory agents from Cordyceps sinensis. Life Sci..

[56] Kuo Y.C., Lin C.Y., Tsai W.J., Wu C.L., Chen C.F., Shiao M.S. (1994). Growth Inhibitors Against Tumor Cells in Cordyceps sinensis Other than Cordycepin and Polysaccharides. Cancer Investig..

[57] Manabe N., Azuma Y., Sugimoto M., Uchio K., Miyamoto M., Taketomo N., Tsuchita H., Miyamoto H. (2000). Effects of the mycelial extract of cultured Cordyceps sinensis on in vivo hepatic energy metabolism and blood flow in dietary hypoferric anaemic mice. Br. J. Nutr..

[58] Koh J.H., Kim J.M., Chang U.J., Suh H.J. (2003). Hypocholesterolemic Effect of Hot-Water Extract from Mycelia of Cordyceps sinensis. Boil. Pharm. Bull..

[59] Chiou W.F., Chang P.C., Chou C.J., Chen C.F. (2000). Protein constituent contributes to the hypotensive and vasorelaxant activities of Cordyceps sinensis. Life Sci..

[60] Winther B., Hoem N., Berge K., Reubsaet L. (2011). Elucidation of phosphatidylcholine composition in krill oil extracted from Euphausia superba. Lipids.

[61] Dawczynski J.S., Jentsch D., Schweitzer M., Hammer G., Strobel L. (2013). Long term effects of lutein, zeaxanthin and omega-3-LCPUFAs supplementation on optical density of macular pigment in AMD patients: The LUTEGA study. Graefes Arch. Clin. Exp. Ophthalmol..

[62] Nestel P., Clifton P., Colquhoun D., Noakes M., Mori T., Sullivan T., Thomas B. (2015). Indications for Omega-3 Long Chain 3 Polyunsaturated Fatty Acid in the Prevention and Treatment of Cardiovascular Disease. Heart Lung Circ..

[63] Sudheendran S., Chang C.C., Deckelbaum R. (2010). N-3 vs. Saturated fatty acids: Effects on the arterial wall. Prostaglandins, Leukot. Essent. Fat. Acids.

[64] Kusšar A., Zupančič A., Šentjurc M., Baričevicč D. (2006). Free radical scavenging activities of yellow gentian (Gentiana lutea L.) measured by electron spin resonance. Hum. Exp. Toxicol..

[65] Mustafa A.M., Caprioli G., Ricciutelli M., Maggi F., Marín R., Vittori S., Sagratini G. (2015). Comparative HPLC/ESI-MS and HPLC/DAD study of different populations of cultivated, wild and commercial Gentiana lutea L.. Food Chem..

[66] Davydov M., Krikorian A.D. (2000). Eleutherococcus senticosus (Rupr. & Maxim.) Maxim. (Araliaceae) as an adaptogen: A close look. J. Ethnopharmacol..

[67] Wagner H., Ulrich-Merzenich G. (2009). Synergy research: Approaching a new generation of phytopharmaceuticals. Phytomedicine.

[68] Da Silva K.R., De Mendonça V.R.R., Silva K.M., Nascimento L.F.M.D., Mendes-Sousa A.F., De Pinho F.A., Barral-Netto M., Barral A.M.P., E Cruz M.D.S.P. (2017). Scoring clinical signs can help diagnose canine visceral leishmaniasis in a highly endemic area in Brazil. Memórias Instituto Oswaldo Cruz.

[69] Maroli M., Rossi L., Baldelli R., Capelli G., Ferroglio E., Genchi C., Gramiccia M., Mortarino M., Pietrobelli M., Gradoni L. (2008). The northward spread of leishmaniasis in Italy: Evidence from retrospective and ongoing studies on the canine reservoir and phlebotomine vectors. Trop. Med. Int. Heal..

[70] An Eys Guillaume J.J.M., Schoone G.J., Kroon N.C., Ebeling S.B. (1992). Sequence analysis of small subunit ribosomal RNA genes and its use for detection and identification of Leishmania parasites. Mol. Biochem. Parasitol..

[71] Inokuma H., Ohno K., Onishi T., Raoult D., Brouqui P. (2001). Detection of Ehrlichial Infection by PCR in Dogs from Yamaguchi and Okinawa Prefectures, Japan. J. Veter-Med Sci..

[72] Biller B., Elmslie R., Burnett R., Avery A., Dow S. (2007). Use of FoxP3 expression to identify regulatory T cells in healthy dogs and dogs with cancer. Veter-Immunol. Immunopathol..

[73] Alfinito F., Ruggiero G., Sica M., Udhayachandran A., Rubino V., Della Pepa R., Palatucci A.T., Annunziatella M., Notaro R., Risitano A.M. (2012). Eculizumab treatment modifies the immune profile of PNH patients. Immunobiol..

[74] Olsen I., Sollid L.M. (2013). Pitfalls in determining the cytokine profile of human T cells. J. Immunol. Methods.

[75] Ferrer L., Aisa M., Roura X., Portús M. (1995). Serological diagnosis and treatment of canine leishmaniasis. Veter-Rec..

[76] Solano-Gallego L., Riera C., Roura X., Iniesta L., Gallego M., Valladares J.E., Fisa R., Castillejo S., Alberola J., Ferrer L. (2001). Leishmania infantum-specific IgG, IgG1 and IgG2 antibody responses in healthy and ill dogs from endemic areas. Veter-Parasitol..

[77] Rodríguez A., Solano-Gallego L., Ojeda A., Quintana J., Riera C., Gállego M., Portús M., Alberola J., Riera M.C. (2006). Dynamics ofLeishmania-Specific Immunoglobulin Isotypes in Dogs with Clinical Leishmaniasis before and after Treatment. J. Veter-Intern. Med..

[78] Nathwani R.A., Pais S., Reynolds T.B., Kaplowitz N. (2005). Serum alanine aminotransferase in skeletal muscle diseases. Hepatology.

[79] Clementi A., Battaglia G., Floris M., Castellino P., Ronco C., Cruz D.N. (2011). Renal involvement in leishmaniasis: A review of the literature. NDT Plus.

[80] Gowda S., Desai P.B., Kulkarni S.S., Hull V.V., Math A.A., Vernekar S.N. (2010). Markers of renal function tests. N. Am. J. Med. Sci..

[81] Jordan J., Sullivan A., Lee T. (2008). Immune Activation by a Sterile Aqueous Extract of Cordyceps Sinensis: Mechanism of Action. Immunopharmacol. Immunotoxicol..

[82] Shi B., Wang Z., Jin H., Chen Y.W., Wang Q., Qian Y. (2009). Immunoregulatory Cordyceps sinensis increases regulatory T cells to Th17 cell ratio and delays diabetes in NOD mice. Int. Immunopharmacol..

[83] Chen J.L., Chen Y.C., Yang S.H., Ko Y.F., Chen S.Y. (2009). Immunological alterations in lupus-prone autoimmune (NZB/NZW) F1 mice by mycelia Chinese medicinal fungus Cordyceps sinensis-induced redistributions of peripheral mononuclear T lymphocytes. Clin. Exp. Med..

[84] Nastasijevic B., Lazarević-Pašti T., Dimitrijević-Branković S., Pašti I., Vujačić A., Joksić G., Vasic V. (2012). Inhibition of myeloperoxidase and antioxidative activity of Gentiana lutea extracts. J. Pharm. Biomed. Anal..

